# The Effect of Phenolic Acids on the Sorption and Wetting Properties of Apple Pectin-Based Packaging Films

**DOI:** 10.3390/molecules30091960

**Published:** 2025-04-28

**Authors:** Magdalena Mikus, Sabina Galus

**Affiliations:** Department of Food Engineering and Process Management, Institute of Food Sciences, Warsaw University of Life Sciences, 159c Nowoursynowska St., 02-776 Warsaw, Poland; magdalena_mikus@sggw.edu.pl

**Keywords:** edible films, apple pectin, phenolic acids, sorption, contact angle

## Abstract

In this article, the effects of different phenolic acids, such as ferulic, gallic, caffeic, coumaric, protocatechuic, and sinapic, as active compounds on the sorption and wetting properties of apple pectin-based edible films were evaluated. The control pectin films and those with added phenolic acids differed in appearance and physical properties. The water content of the films was reduced and ranged from 8.91 ± 0.01% to 13.44 ± 0.01% for films containing phenolic acids compared to the control films (14.31 ± 0.01%). The swelling index value of the films ranged from 86.63% for films with protocatechuic acid to 88.33% for films with the addition of caffeic acid. It was observed that the sorption isotherms had a similar shape for all the obtained films, while scanning electron microscopy (SEM) allowed for the observation of changes in the structure resulting from the film composition. It was shown that the lowest water contact angle values at the initial time (0 s) were observed for pectin films with ferulic acid (47.00° ± 4.47) and the highest for the control films (58.44° ± 5.62). After 60 s, the highest water contact angle value was recorded for the film with caffeic acid (66.39° ± 5.18) and the lowest for the film with ferulic acid (14.72° ± 5.70). Films containing gallic acid and protocatechuic acids showed the lowest water vapour permeability values among active films. The edible films developed in this study showed desirable features that could be used as bioactive packaging for food industry applications, both as protective edible coatings and active packaging films.

## 1. Introduction

Packaging is necessary to maintain the organoleptic, nutritional, and hygienic properties of food products. Due to the wide variety of packaging films, they can be classified as synthetic or biodegradable [1,2]. Nowadays, films made from plastics very often constitute an environmental problem [3]. Therefore, more and more often, research is being conducted to find alternative materials that will reduce the consumption of films made from non-renewable raw materials and replace synthetic polymers [4,5]. Their use in the food sector can reduce environmental problems related to accumulating non-renewable synthetic materials [6]. In addition, the use of edible coatings provides protection of the product against mechanical damage during transport, handling, and storage [7], thus ensuring an extension of shelf life, with no or minimal noticeable impact on the texture, taste, and nutritional value of the food [8]. This form of edible packaging is an edible coating, a thin layer of material placed on the surface of food products. The coating is a semi-permeable barrier to gases and affects the integrity of the food [9]. In addition, the coating can be a carrier for bioactive compounds that act on the surface of food or in the human intestines, which include probiotics, antimicrobials, antivirals, and antioxidants. On the other hand, an edible film is a layer of material formed outside the food product, which is then applied to the product [10].

Edible films can be prepared based on proteins, polysaccharides, lipids, or a combination of these materials. Proteins and polysaccharides are characterised by their ability to create coatings with good oxygen barrier properties but are a poor moisture barrier [11]. In addition, films made based on polysaccharides are characterised by good permeability to carbon dioxide and have appropriate optical properties. They can be transparent and do not cause changes in the taste and smell of food [12].

Pectin is a water-soluble polysaccharide that consists mainly of linked esterified units of α-d-galacturonic acid. It is characterised by a high molecular weight and the ability to form a firm gel, e.g., in the presence of calcium cations [13]. Pectin is one of the main components of plant cell walls and has different structures depending on the source [14].

Pectin can be extracted from food industry waste, particularly apple pomace, transforming what would otherwise be a throwaway by-product into valuable packaging materials. Furthermore, advances in pectin extraction and the use of state-of-the-art techniques such as supercritical water extraction, ultrasound, or microwave-assisted extraction are improving the quality and functionality of the resulting material, leading to new possibilities in food packaging. Due to its biodegradability, edibility, and ability to form films in combination with other materials, pectin is an attractive option for developing environmentally friendly alternatives to non-biodegradable plastics. Films based on pectin provide a barrier against oxygen, making them suitable for packaging fresh fruits. They are also characterised by their ability to limit respiration and extend the shelf life of perishable products [15]. Pectin-based films are also resistant to oils and fats, dissolve in water, and have excellent mechanical properties [16]. However, it should be noted that pectin films, without adding any other polymers, are characterised by high water permeability and high water solubility and have limited use in high-humidity atmospheres or the case of packaging food products with high moisture content [17]. Various modifications are used to improve these properties and increase the use of pectin-based films [18,19].

One of the modifications used is adding phenolic compounds to edible films, which provide antioxidant properties. This innovative approach, gaining popularity worldwide to obtain active packaging, can improve food products’ oxidative state and antimicrobial properties. Another advantage of phenolic compounds is that they are natural and bioactive compounds found in various food products, e.g., fruits, vegetables, herbs, oils, and spices [20]. In addition, their occurrence has also been noted in agricultural and industrial by-products. Phenolic acids are defined as non-flavonoid polyphenolic substances, the characteristic feature of which is the connection of a carboxyl group with a benzene ring. They are a desirable group of compounds in the food industry because they inhibit harmful bacteria and fungi, including *E. coli*, *Bacillus cereus*, *Staphylococcus aureus*, or *Aspergillus flavus* [21].

For example, phenolic acids, including ferulic acid, which is one of the most common acids in plants, are added to improve various properties. In addition to low toxicity and good cross-linking properties, ferulic acid has several other desirable properties, such as antioxidant, antimicrobial, anti-inflammatory, or antithrombotic effects. In addition, it is characterised by anticancer effects, protects against coronary artery disease, and increases sperm viability. It has been shown that in addition to ferulic acid, caffeic and gallic acids exhibit cross-linking effects [22]. Tannic acid also has excellent antioxidant, antimicrobial, and antiviral effects. In addition, it is considered safe in the food sector and prevents food spoilage and the development of pathogenic microorganisms [23].

The main objective of this study was to evaluate the sorption and wetting properties of active films based on apple pectin containing phenolic acids. In addition, it was determined which phenolic acid had the greatest impact on the properties of the films and their potential use as an edible coating or packaging film for food applications. The studies included thickness, water content, the swelling index, water solubility, water vapour sorption kinetics, water vapour sorption isotherms, water contact angle, the morphology of film measurements, and the water vapour permeability of active pectin films.

## 2. Results

### 2.1. Film Characterisation

**Figure 1:**
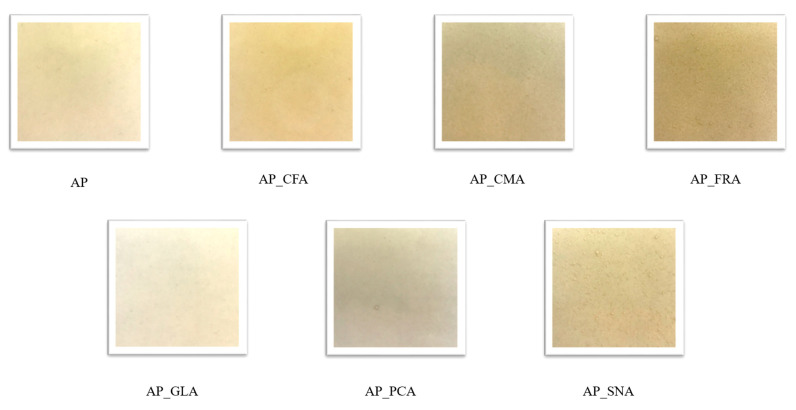
Photographs of the apple pectin (AP) edible films obtained with the addition of caffeic (CFA), coumaric (CMA), ferulic (FRA), gallic (GLA), protocatechuic (PCA), and sinapic (SNA) acids.

An essential role in the context of considering edible films as packaging materials is played by their physicochemical properties. A smooth, uniform structure characterised the produced pectin films with the addition of selected phenolic acids. Photographs of the prepared films are presented in Figure 1. All the films obtained were transparent or slightly yellow to slightly brown. The addition of a plasticiser positively affected the obtained films by increasing their plasticity.

Thickness, moisture content, and the swelling index are important characteristics concerning using edible films as food packaging materials. Considering the thickness parameter, it was observed that the control films characterised the lowest value for this parameter. In contrast, the highest was characterised by the films adding coumaric acid. A similar relationship was observed by Yang et al. [24], who studied the thickness of films obtained from pectin and with the addition of tannic acid and Fe^3+^. The thickness of the control pectin film was 86 µm, while it increased with the addition of tannic acid and Fe^3+^, reaching a maximum value of 97 µm. The increase in the solid content and disruption of the original structure of the film probably caused this increase. According to Yerramathi et al. [25], the thickness of the film most often depends on the dynamics of film dispersion during preparation and drying, which later affects the achieved physical and mechanical properties of edible composite films. The pectin films obtained by Liu et al. [26] reached a thickness of 66 ± 3.61 µm, while a thickness of 68.67 ± 2.40 µm characterised the films with the addition of gallic acid.

The water content indicates the water retention capacity of the produced film matrix. The results of the water content of the tested films are presented in Table 1. The values were in the range of 8.91–14.31%. These values are similar to other biopolymeric films obtained via casting [27,28,29] or electrospinning methods [30]. The highest water content was observed for the control film, while the lowest was obtained with the addition of sinapic acid. This could be attributed to the film structure and the formation of bonding in the film matrix that probably affected the higher rate of water evaporation when sinapic acid was added. Edible films containing high moisture content and water solubility are unsuitable packaging materials for food products requiring high water resistance. The water content of pectin films was reduced after adding phenolic acids due to many reasons, mostly due to differences in compatibility between acids and biopolymers, the acid distribution in the film matrix due to the various rates of solubility, film structure creation during storage (evaporation of water), and the film thickness.

### 2.2. The Effect of Phenolic Acids on the Swelling Index of Apple Pectin Edible Films

The swelling parameter of the control and pectin films with added phenolic acids after being placed in a beaker with distilled water was also analysed (Table 1). The solubility parameter allows for determining the extent to which the edible film can act as a barrier and provide resistance to moisture. The swelling index indicates the film’s ability to retain water resulting from a hydrophilic group in its structure in the film matrix. Groups that easily interact with water include the hydrophilic and hydroxyl groups [31]. A moment after being placed in water, the apple pectin films and those with added gallic, coumaric, and sinapic acids began to dissolve in water and lose their integrity. This phenomenon can be attributed to the hydrophilic hydroxyl groups present in the structure of the films. Pectin films dissolve in water due to the pectin structure’s hydroxyl and non-esterified carboxyl functional groups, which can form hydrogen bonds [32]. The control films obtained without the addition of phenolic acids dissolved the fastest after being placed in distilled water (100%), which could be due to the good solubility properties of the film and biodegradability. In addition, it was found that the use of a plasticiser, glycerol, had no effect on the water solubility parameter because the same amount was used in each variant. The addition of ferulic, caffeic and protocatechuic acids influenced the stability of the film after being placed in water, which meant that they did not decompose. The lower water solubility of the film may also be due to the formation of long-chain molecules with low water solubility. The highest value of the swelling index in water was recorded for the film with the addition of caffeic acid (88.33 ± 1.06%). Phenolic acids contain many hydroxyl groups that are capable of binding water. According to reports by Rahmawati et al. [33], adding more gallic acid reduces the thickness and water solubility of edible films. Polysaccharides and phenolic compounds are characterised by their easy formation of complexes in food systems, which are usually driven mainly by hydrogen bonds and, to a lesser extent, by hydrophobic interactions, affecting phenolic compounds’ chemical stability and bioavailability. In addition, gallic acid has a hydrophobic nature, which may result in film swelling [31].

### 2.3. The Effect of Phenolic Acids on the Water Vapour Sorption Kinetics

**Figure 2:**
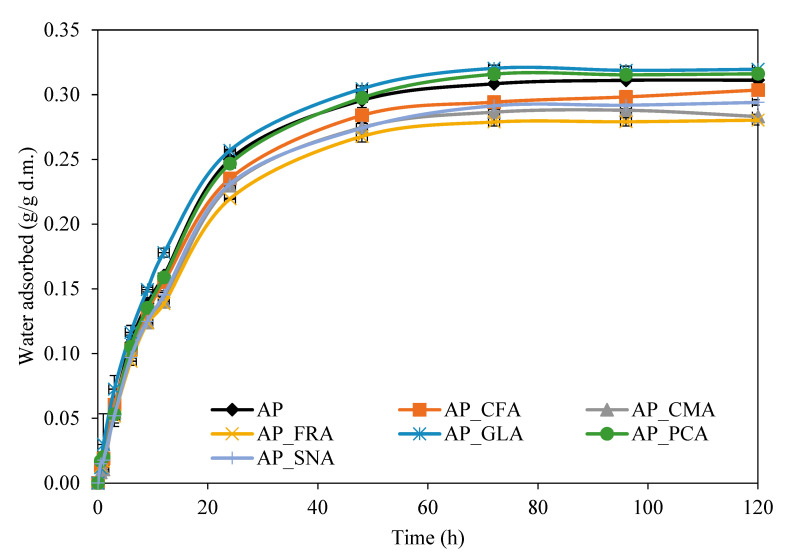
Water adsorbed by edible films for the apple pectin (AP) edible films obtained with the addition of caffeic (CFA), coumaric (CMA), ferulic (FRA), gallic (GLA), protocatechuic (PCA), and sinapic (SNA) acids.

The water vapour transport rate through edible films depends on the adsorption, diffusion, and desorption rates. This phenomenon involves water molecules dissolving on one side of the film, moving into the empty space between polymer segments, and then desorbing from the polymer surface on the other side of the edible film [31]. The physicochemical properties of the films largely depend on the intermolecular interactions occurring in the film matrix. Figure 2 shows the kinetics of the water vapour sorption of the film. The highest water adsorption value was observed for the pectin film with the addition of gallic acid (0.320 ± 0.001 g/g d.m.), and the lowest was for the film obtained with ferulic acid (0.280 ± 0.004 g/g d.m.). All the analysed film variants reached equilibrium within 24 h. Moreover, the obtained films’ water vapour sorption kinetics curves were characterised by a similar shape and course. In the initial phase, all the films did not differ significantly in water content, which allows us to state that the driving force of the sorption process was similar and was most intensive during the initial 10 h.

The barrier properties of films are mainly influenced by the materials from which the edible films are obtained, the additives, and the film preparation process itself. According to Cheng et al. [34], adding phenolic acids to the film matrix reduces the films’ water vapour permeability (WVP). Moreover, it was found that this tendency is dominant at low concentrations because the presence of hydrophobic phenolic compounds limits the diffusion of water through the matrix.

The values of the water vapour diffusion coefficient in edible films are shown in Table 2. It was observed that adding phenolic acids to film-forming solutions influenced the increase in the water vapour diffusion coefficient. The values ranged from 0.87 ± 0.04 × 10^−14^ m^2^/s for control films (AP) to 2.80 ± 0.18 × 10^−14^ m^2^/s for films containing phenolic acids. The lowest increase occurred for films with the addition of protocatechuic acid (0.88 ± 0.03 × 10^−14^ m^2^/s), while the highest was for coumaric and ferulic acids. Ordoñez et al. [35] found that the active compound’s diffusion coefficient and release percentage highly depend on the polarity of the food substrates and the polymer matrix. This is due to different molecular interactions and the chemical affinity between the active compound, the polymer matrix, and food substrates. Various values may also result from differences in the molecular weight of the compound and its structure, the microstructure of the film, and the strength of the compound’s bond in the matrix. In addition, the factors influencing the value of the diffusion coefficient are the compound’s solubility and the interactions between the substance and the polymer, which affect the modification of the cohesive forces of the polymer chain.

### 2.4. The Effect of Phenolic Acids on the Water Vapour Sorption Isotherms

Water sorption isotherms are widely used to express the relationship between moisture content and water activity for various materials. In the case of hydrophilic biopolymer films, this is of great importance because the characteristics of such films indicate the sensitivity of the films to water, which is tantamount to the existence of a relationship between their functionality and water [36]. Isotherms can be represented as adsorption or desorption isotherms. Adsorption isotherms are obtained by measuring the increase in weight due to moisture uptake. This phenomenon occurs when a completely dry material is placed in an environment of increasing relative humidity. Desorption isotherms are obtained when a wet material is placed in an environment of constant relative humidity. In the case of adsorption isotherms, five types are distinguished, which depend on the shape of the curve and the process [37].

**Figure 3:**
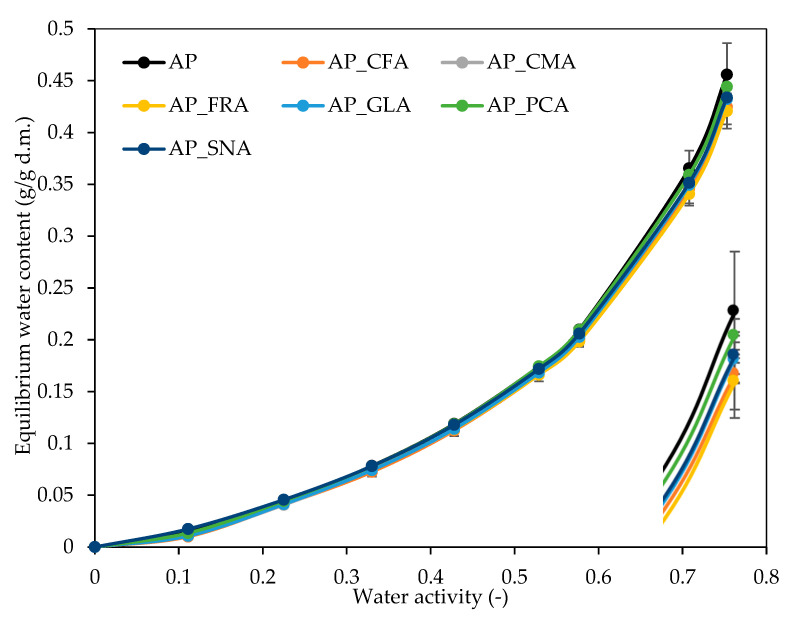
Water vapour sorption isotherms of the apple pectin (AP) edible films obtained with the addition of caffeic (CFA), coumaric (CMA), ferulic (FRA), gallic (GLA), protocatechuic (PCA), and sinapic (SNA) acids.

Moisture adsorption is a very important indicator that determines the sensitivity of the material to moisture. Figure 3 shows the water vapour sorption isotherms for the control film and with the addition of selected phenolic acids. The shape of the isotherms for pectin films with phenolic acids was smaller at lower water activity, and with each increase in water activity, there was an increase in relative humidity. In addition, the sorption process was most intensive in the initial 10 h. The isotherms shown in Figure 3 represent a type of isotherms called Flory–Huggins isotherms, which are characterised by convexity at all points. According to Nazreen et al. [38], this type of graph occurs due to the weak interaction between the adsorbent surface and the adsorbate, which is necessary to determine the stability of the film packaging. This type of isotherms is often assigned to edible films, e.g., chitosan films. The determination of the film’s sensitivity to moisture and the type of environment is necessary to maintain the shelf life of the package, as well as the quality of the food product and the stability during transport and storage. Furthermore, the isothermal moisture sorption of the material represents the predicted amount of water that will be retained in the material under specific conditions of relative humidity and temperature.

Also, Veras et al. [36] obtained similar shape sorption isotherms of pectin films with propolis. It was observed that the equilibrium water content in the film decreased with increasing temperature. According to Othman et al. [39], higher temperatures are closely related to lower water content in the film in the monolayer, which results in fewer sorption sites. Therefore, it is stated that storing the film at high temperatures and relative humidity reduces the possibility of avoiding autoxidation. This increases the probability that the edible film will be characterised by instability during storage.

### 2.5. The Effect of Phenolic Acids on the Water Contact Angle

In the case of packaging materials, resistance to water absorption is a very important property. The hydrophilicity of edible films can be determined based on the water contact angle of the film surface layer, which also provides information on the interactions occurring at the phase boundary. The results of water contact angles for edible films are presented in Table 3. It is stated that surfaces with a water contact angle of less than 90° are hydrophilic, while surfaces with a water contact angle greater than 90° are characterised by hydrophilicity and complete or partial wetting [40].

As can be seen from Table 3, the initial water contact values for the films ranged from 47.00° ± 4.47 to 58.44° ± 5.62. After 60 s, the most hydrophobic surface was observed in films with the addition of caffeic acid (60.92° ± 4.93) and protocatechuic acid (56.42° ± 4.27). This may be due to the increased hydrophobicity of the film due to the strengthening of the structure and the occurrence of intermolecular interactions between the matrix and caffeic acid. In addition, the water contact angle can also be related to the occurrence of slight surface roughness of the film. This statement results from the experiments which showed that the water contact angle depends not only on the interfacial energies but also on the surface structure, its pretreatment, and the existing contaminants [41]. According to Tavassoli-Kafran et al. [42], a high affinity for water characterises hydrophilic surfaces with a water contact angle of less than 90°. According to Żelaziński [43], the most hydrophobic materials defined as biodegradable can achieve a water contact angle of up to 158°. It is also worth noting that pure polylactic acid (PLA) has a water contact of 75°.

### 2.6. The Effect of Phenolic Acids on the Microstructure of Pectin Films

Microstructure is a key parameter for controlling the mechanical and barrier properties of edible films and is essential for understanding the fundamentals of materials science from a practical point of view [44]. Scanning electron microscopy (SEM) is performed to determine the morphological features of the surface and the cross-sections of edible films. The study of the microstructure of films allows for the determination of the influence of modifiers on the structure formation processes, as well as the simultaneous identification of cracks, porosity, roughness, or homogeneity of edible films. SEM is recommended for studying the microstructure primarily in composite films, nanoemulsions, or when using functional additives, e.g., nanoparticles [45]. In addition, the microstructure of edible films depends on how the edible films are prepared, the ingredients used, and the interactions between the plasticiser and the components [7].

**Figure 4:**
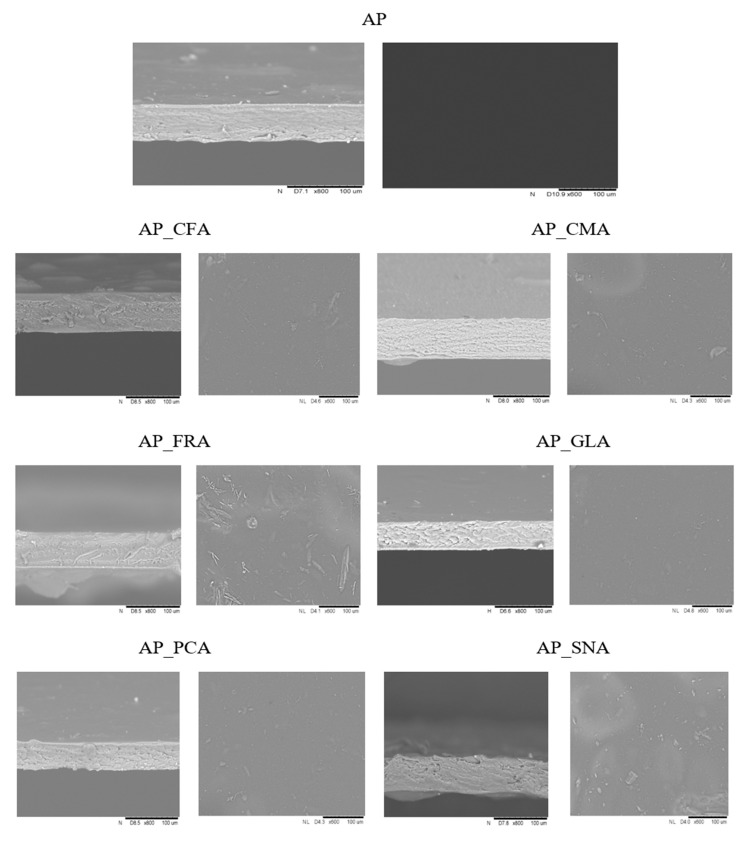
Photographs of cross-sections and surfaces of edible films for the apple pectin (AP) edible films obtained with the addition of caffeic (CFA), coumaric (CMA), ferulic (FRA), gallic (GLA), protocatechuic (PCA), and sinapic (SNA) acids. Magnifications: 600× (cross-sections) and 800× (film surfaces).

The observation of the surface (600×) and cross-sections (800×) of the obtained pectin films with the addition of selected phenolic acids was carried out using scanning electron microscopy (SEM) (Figure 4). Based on different additions of phenolic acids, the obtained films can have different structures. No cracks were found in the cross-section. Microphotographs of the control films and those with the addition of phenolic acids are smooth, uniform, and compact, which confirms their high compatibility, good adhesion, and integrity between them. The layer adjacent to the sheet of the obtained films was shiny, while the other side was more matt. This may be due to the drying conditions or the addition of various types of phenolic acids. The control film showed a smooth and uniform structure on the outer and substrate sides.

The smooth surface of the film indicates good compatibility between pectin and phenolic acids. According to Yerramathi et al. [25], controlled cross-linking of ferulic acid results in an edible biopolymer with a homogeneous and stable structure, which is used for preserving food products and other production applications. In addition, the studies conducted by Yong et al. [46] show that the hydroxyl groups present in hydroxycinnamic acids (e.g., p-coumaric acid, caffeic acid, and ferulic acid) can interact with chitosan skeletons and glycerol through the hydrogen bonds that occur, ensuring a homogeneous structure of the film. The tested caffeic acid had two hydroxyl groups, allowing it to interact with chitosan skeletons and the plasticiser (glycerol). Adding phenolic acids to the film matrix reduces the roughness parameters of the pectin films [47].

### 2.7. The Effect of Phenolic Acids on the Water Vapour Permeability of Pectin Films

In general, when applied to food products, the main function of an edible film or coating is to reduce moisture transfer between the coated food and the surrounding atmosphere or between two components of a heterogeneous food product. Therefore, it is essential that the water vapour permeability is as low as possible. The results for the water vapour permeability of the developed active packaging films are presented in Table 4. The values ranged from 7.16 to 10.48 × 10^−10^ g/m·s·Pa. A decrease in water vapour barrier efficiency can be observed due to the addition of phenolic acids. However, the values were similar for films containing gallic and protocatechuic (×10^−10^ g/m·s·Pa) acids when compared to the control films (7.45–7.46 × 10^−10^ g/m·s·Pa), and the differences were not statistically significant (*p* < 0.05). On the other hand, the rest of the films revealed significantly higher water vapour permeability values of 9.76 × 10^−10^ g/m·s·Pa for caffeic acid and 10.45–10.68 ×10^−10^ g/m·s·Pa for films containing other phenolic acids. This was probably attributed to the differences in compatibility between acids and biopolymers, the acid distribution in the film matrix due to the various rates of solubility, film structure creation during storage (evaporation of water), and the film thickness. Moreover, the hydrophilicity of the plasticiser (glycerol used in this study) is one of the main factors that affect water vapour permeability. Glycerol causes mobility in the film matrix by reducing intermolecular forces and creating free volume. In this context, the motion of water vapour becomes easier in biopolymer-based materials.

The obtained results of water vapour permeability of pectin films containing phenolic acids are similar to other biopolymeric films. However, the values vary due to biopolymer type or different methods used for film preparation, as well as differences in methods conducting water vapour permeability test. Thus, various conditions (temperature or humidity differentials in the gravimetric method) are also used. Hager et al. noted that the addition of tannic acid (1–30%) could potentially decrease the water vapour permeability of wheat gluten films. The authors explained that the improvement of barrier properties is probably related to the tight gluten−tannic acid network, which likely occupies previously hydrophilic hydroxyl groups and traps small air bubbles inside the matrix, resulting in reduced water molecule sorption in the cross-linked film. On the other hand, for wheat gluten films containing gallic acid, an increase in values at lower concentrations (1–2%) followed by a decrease at higher concentrations (5–10%) was observed [48]. The same tendency phenomenon was observed for chitosan films incorporated with gallic acid [49]. Similar observations were noted by Yang et al. [24]. The addition of tannic acid to pectin films resulted in a decrease in the water vapour transmission rate, which the authors attribute to the crosslinking between tannic acid and pectin, leading to a denser network structure within the pectin film and consequently reducing the water vapour permeability.

## 3. Materials and Methods

### 3.1. Materials

The research material consisted of edible films produced based on apple pectin (Pektowin S.A., Jasło, Poland), selected phenolic acids (POL-AURA) (Warsaw, Poland), such as ferulic acid, gallic acid, caffeic acid, coumaric acid, protocatechuic acid (Thermo Scientific) (Gdańsk, Poland), and sinapic acid (Acros Organics) (Poznań, Poland). Glycerol (Avantor Performance Materials, Gliwice, Poland) was a plasticising agent.

### 3.2. Film Preparation

**Figure 5:**
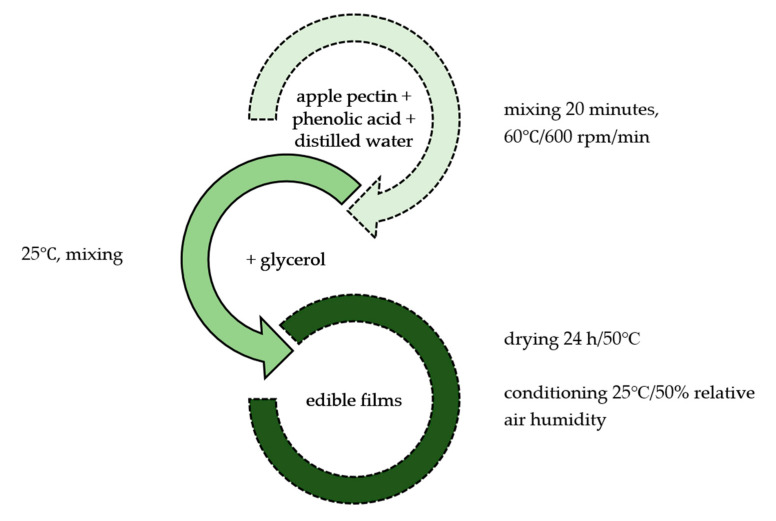
Scheme of film preparation.

The film-forming preparation steps are presented in Figure 5. Aqueous film-forming solutions were prepared with a concentration of 5% apple pectin, selected phenolic acids (5%), and the addition of a plasticiser (50% in relation to the weight of apple pectin).

The solutions were mixed for 20 min using an RCT basic IKAMAG hot plate (60 °C) (IKA Werke Gmn & Co., Staufen, Germany) and an RCT basic IKAMAG magnetic stirrer (IKA Poland, Warsaw, Poland) rotating at a speed of 600 rpm. The film-forming solutions were poured onto sheets at 10 mm/s and a layer thickness of 2500 µm using a Zehntner ZAA 2300 automatic film applicator (Zehntner GmbH Testing Instruments, Sissach, Switzerland). The films were dried in a SUP-65W laboratory dryer (Wamed, Warsaw, Poland) for 24 h at 50 °C. Then, the obtained films were conditioned in a KFB 240 thermostatic chamber (Binder, Tuttlingen, Germany) at 25 °C and 50% relative air humidity for 48 h prior to testing.

### 3.3. Thickness

The thickness was determined using a thickness tester (Thwing-Albert, ProGage Thickness Tester, West Berlin, NJ, USA) with an accuracy of 1 μm. The thickness of the films was measured for each experiment at least in 3 repetitions.

### 3.4. Water Content

The determination of the water content in the edible films was carried out by drying samples in a laboratory dryer (SUP 65 WG, WAMED, Warsaw, Poland) at 105 °C for 24 h with an accuracy of ±0.0001 g using an analytical balance (RADWAG PS 600/C/2, Radom, Poland). Measurements were made in 3 repetitions, and the dry matter was calculated according to the following equation:

$$d.m.=\frac{m_{s}-m}{m_{p}-m}\cdot 100\%$$

where $m_{s}$ is the sample weight after drying (g), $m_{p}$ is the sample weight before drying (g), and m is the mass of the empty weighing vessel (g).

### 3.5. Swelling Index

The prepared foils in squares with dimensions of 2 × 2 cm were weighed on an analytical balance with an accuracy of ±0.0001 g; then, the samples were placed in 25 mL of distilled water for 2 min. Then, the films were filtered to remove excess water using filter paper and weighed again. Each measurement was performed in 3 repetitions. The swelling of the foil was calculated based on the following formula [50]:

$$P=\frac{m_{2}-m_{1}}{m_{1}}\cdot 100\%$$

where

P is the swelling of the edible films (%);
$m_{1}$ is the sample weight before swelling (g);
$m_{2}$ is the sample weight after swelling (g).

### 3.6. Water Solubility

The films in squares with 2 × 2 cm sides were placed in glass vessels, weighed on an analytical balance with an accuracy of ±0.0001 g, and dried for 24 h at 105 °C. After this time, the samples were cooled in a silica gel desiccator. The films were weighed again and placed in 25 mL of distilled water. After 24 h of storage and occasional mixing, excess water was removed using filter paper. The films were again placed in the laboratory dryer for 24 h at 105 °C and weighed. The measurement was performed in 3 repetitions, and the solubility in water (*R*) was determined based on the following formula [51]:

$$R=\frac{m_{0}-m_{r}}{m_{0}}\times 100\%$$

where $m_{0}$ is the dry mass of the sample before dissolving (g) and $m_{r}$ is the dry mass of the sample after dissolving (g).

### 3.7. Water Vapour Sorption Kinetics

The water vapour sorption kinetics was determined based on the change in mass of the film samples with a mass of ±0.25 g and with an accuracy of ±0.0001 g (Radwag, Radom, Poland), with weighing conducted over a period of time. The determination was performed at a relative humidity of 100% (distilled water) at times 0, 0.5, 1, 3, 6, 9, 12, 24, 48, 72, 96, and 120 h. Based on the obtained kinetic curves, the results of water vapour sorption kinetics were interpreted, which were graphs of the dependence of the change in the amount of water that was adsorbed (g/g dry matter) on the time of the process (h). Using Fick’s second law, the diffusion coefficient (D) of water vapour was calculated based on the following formula:

$$\frac{M_{t}-M_{0}}{M_{e}-M_{0}}=1-\sum_{n=0}^{\infty }\frac{8}{{\left(2+1\right)}^{2}\pi^{2}}exp\left[-\frac{{\left(2\pi +1\right)}^{2}\pi^{2}}{{4L}^{2}}Dt\right]$$

where $M_{t}$ is the total amount of adsorbed water (g/g dry matter) during the process (s), $M_{0}$ is the initial water content (g/g d.m), $M_{e}$ is the water content over time *t* (g/g d.m), and L is the film thickness (m).

### 3.8. Water Vapour Sorption Isotherms

The water vapour adsorption isotherms of the coatings were determined in 2 repetitions using the dynamic water vapour sorption apparatus Aquadyne DVS-2HT (Quantachrome Instruments by Anton Paar Sp. z o.o., Warsaw, Poland) in the range of environmental relative humidities from 0 to 75%. Experimental data points were analysed using Microsoft Excel 2020 and aquaWIN Software (latest version Air3). The water vapour adsorption isotherms were presented as curves of the dependence of equilibrated water content on water activity.

### 3.9. Water Contact Angle Measurement

The water contact angle analysis was performed using the drop-by-drop method with an OCA 25 goniometer (DataPhysics Instruments, Filderstadt, Germany). The contact angle was measured after applying a 10 μL drop of distilled water at a rate of 10 μL/s to the film surface. The analysis was performed in at least 6 repetitions at 0 and 60 s, and the results were processed using the SCA20_U software (Version 5.0.37).

### 3.10. Scanning Electron Microscopy

The observations of the cross-sections’ structure and the films’ surface were made using a TM3000 table scanning electron microscope (Hitachi High Tech, Tokyo, Japan). The 5 × 5 mm films were placed on the measuring table using the carbon paste PELCO with a diameter of 9 mm (Pik Instruments Sp. z o.o., Piaseczno, Poland). The measurement was carried out in a low vacuum condition of 0.35–1 torr with the cross-sections magnified at 800× and the surface magnified at 600×.

### 3.11. Water Vapour Permeability

A gravimetric method was used to evaluate the water vapour permeability of the analysed films using the Mater Cup FX-3180 equipment (Textest AG, Schwerzenbach, Switzerland). Three samples were cut from each film, and their thickness was measured. The samples were placed between two rubber-based rings on top of cells containing distilled water, using a relative humidity gradient of 50–100% and a permeation surface of 28.3 cm^2^.

### 3.12. Statistical Analysis

Statistical analysis was performed using the Statistica 13.3 programme by analysing the variance in the system with repeated measurements and one-way analysis of variance (ANOVA) with the Tukey post hoc test, with a significance level 0.05.

## 4. Conclusions

The studies confirmed that phenolic acids are effective cross-linking agents for materials based on apple pectin. The analysis of the thickness and water content of the films showed that the addition of phenolic acids increased their thickness and reduced the water content. In addition, the sorption process was most intensive in the first 10 h and was similar for all film variants. Films obtained based on apple pectin modified with caffeic acid are promising in terms of potential use as packaging because they achieved the highest values of the water contact angle and, thus, the highest hydrophobicity, limiting water absorption. Using edible films with the addition of caffeic acid could minimise the adverse effect of moisture on the product. On the other hand, films containing gallic acid and protocatechuic acids showed the lowest water vapour permeability values among active films, thus indicating the potential for applications for food products where water barrier efficiency is crucial. Microscopic analysis proved that all the films obtained were smooth and homogeneous, with good compatibility between apple pectin and phenolic acids. The obtained edible films could have potential applications in the food industry, as they prevent changes in colour and texture. However, more research is needed for coated food products or the application of developed films as edible pouches. This need arises from evaluating the mechanical strength and durability of the materials, as well as the compatibility of the edible films with food products.

## Figures and Tables

**Table 1:** The water content, swelling index, and solubility in water for the apple pectin (AP) edible films obtained with the addition of caffeic (CFA), coumaric (CMA), ferulic (FRA), gallic (GLA), protocatechuic (PCA), and sinapic (SNA) acids.

Film Type	Thickness (µm)	Water Content (%)	Swelling Index (%)	Solubility in Water (%)
AP	76.76 ± 5.39 ^a^	14.31 ± 0.01 ^d^	100 ^ab^	77.39 ± 8.00 ^a^
AP_CFA	109.79 ± 7.81 ^b^	10.70 ± 0.01 ^ab^	88.33 ± 1.06 ^ab^	70.75 ± 0.29 ^a^
AP_CMA	129.49 ± 7.37 ^c^	11.36 ± 0.01 ^abc^	100 ^ab^	70.22 ± 0.39 ^a^
AP_FRA	121.88 ± 7.67 ^c^	13.44 ± 0.01 ^cd^	88.18 ± 0.71 ^a^	79.11 ± 7.92 ^a^
AP_GLA	85.31 ± 4.05 ^a^	10.93 ± 0.00 ^abc^	100 ^ab^	69.73 ± 2.91 ^a^
AP_PCA	78.26 ± 5.10 ^a^	12.87 ± 0.01 ^bcd^	86.63 ± 0.24 ^a^	66.26 ± 1.20 ^a^
AP_SNA	112.16 ± 6.07 ^b^	8.91 ± 0.01 ^a^	100 ^ab^	69.11 ± 3.29 ^a^

Mean values ± standard deviations. Different superscript letters (^a–d^) within the same column indicate significant differences between the films (*p* < 0.05).

**Table 2:** Water vapour diffusion coefficient for the apple pectin (AP) edible films obtained with the addition of caffeic (CFA), coumaric (CMA), ferulic (FRA), gallic (GLA), protocatechuic (PCA), and sinapic (SNA) acids.

Film Type	Water Vapour Diffusion Coefficient (×10^−14^ m^2^/s)
AP	0.87 ± 0.04 ^a^
AP_CFA	1.72 ± 0.03 ^c^
AP_CMA	2.80 ± 0.18 ^e^
AP_FRA	2.23 ± 0.13 ^d^
AP_GLA	1.23 ± 0.13 ^b^
AP_PCA	0.88 ± 0.03 ^a^
AP_SNA	1.82 ± 0.08 ^c^

Mean values ± standard deviations. Different superscript letters (^a–e^) within the same column indicate significant differences between the films (*p* < 0.05).

**Table 3:** Water contact angle (θ) results and contact angle photos at 0 and 60 s for the apple pectin (AP) edible films obtained with the addition of caffeic (CFA), coumaric (CMA), ferulic (FRA), gallic (GLA), protocatechuic (PCA), and sinapic (SNA) acids.

Film Type	θ (◦)			
	Time (s)			
	0		60	
AP	58.44 ± 5.62 ^b^	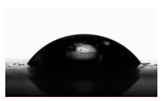	25.71 ± 1.98 ^a^	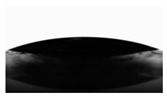
AP_CFA	58.40 ± 4.47 ^b^	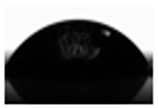	60.92 ± 4.93 ^c^	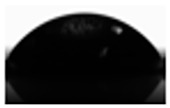
AP_CMA	52.29 ± 2.38 ^ab^	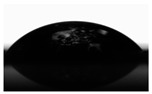	26.88 ± 2.12 ^a^	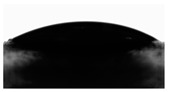
AP_FRA	47.00 ± 4.47 ^a^	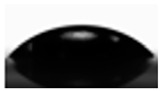	25.03 ± 9.23 ^a^	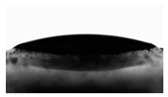
AP_GLA	47.61 ± 4.02 ^a^	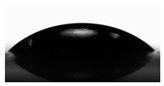	24.81 ± 3.82 ^a^	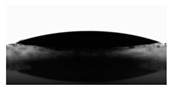
AP_PCA	56.51 ± 5.91 ^b^	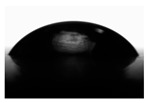	56.42 ± 4.27 ^bc^	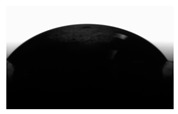
AP_SNA	55.36 ± 2.89 ^b^	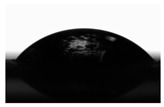	43.11 ± 3.95 ^b^	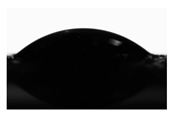

Mean values ± standard deviations. Different superscript letters (^a–c^) within the same column indicate significant differences between the films (*p* < 0.05).

**Table 4:** Water vapour permeability (WVP) of edible films based on apple pectin (AP) with the addition of caffeic (CFA), coumaric (CMA), ferulic (FRA), gallic (GLA), protocatechuic (PCA), and sinapic (SNA) acids.

Film Type	WVP (×10^−10^ g/m·s·Pa)
AP	7.16 ± 0.42 ^a^
AP_CFA	9.76 ± 0.06 ^b^
AP_CMA	10.68 ± 0.12 ^c^
AP_FRA	10.46 ± 0.16 ^c^
AP_GLA	7.45 ± 0.17 ^a^
AP_PCA	7.46 ± 0.22 ^a^
AP_SNA	10.48 ± 0.22 ^c^

Mean values ± standard deviations. Different superscript letters (^a–c^) within the same column indicate significant differences between the films (*p* < 0.05).

## Data Availability

The dataset is available upon request from the authors. The raw data supporting the conclusions of this article will be made available by the authors upon request.
